# Factors Associated with the Severity of ERCP-Related Complications: A Retrospective Single-Centre Study

**DOI:** 10.3390/jcm13237481

**Published:** 2024-12-09

**Authors:** Kristel Goubert, Helena Degroote, Martine De Vos, Maxim Khalenkow, Pieter Hindryckx

**Affiliations:** Ghent University Hospital, Coupure Rechts 136, 9000 Ghent, Belgium; helena.degroote@uzgent.be (H.D.); martine.devos@ugent.be (M.D.V.); maxim.khalenkow@ugent.be (M.K.); pieter.hindryckx@uzgent.be (P.H.)

**Keywords:** endoscopic retrograde cholangiopancreatography, risk factors, adverse effects, anticoagulant therapy, procedural difficulty

## Abstract

**Objectives:** Risk factors for post-endoscopic retrograde cholangiopancreatography (ERCP) complications have been extensively studied and are well established; most complications are mild and self-limiting. This study aims to identify patients at risk of severe early post-ERCP complications. **Methods:** We conducted a retrospective cohort study with data from 2810 ERCP procedures performed at Ghent University Hospital between 2016 and 2022. Patient records and a maintained ERCP registry were used to identify all ERCP-related complications and possible risk factors. The AGREE classification was used to determine the severity of the complication. Univariate and multivariate logistic regression analyses were performed to identify independent predictors of severe complications. **Results:** Out of 2810 procedures, 223 cases (7.9%) had post-ERCP complications, with severe complications occurring in 20.3% of cases. The most common severe complication was haemorrhage (22/49 severe complications, 44.9%), with perforation having the highest probability of being severe (10/15 cases, 67%). Independent predictors of severe complications included anticoagulative therapy (OR 6.3, 95% CI 1.4–28.3, *p* = 0.016) and high procedural difficulty (Schutz category 3: OR 11.5, 95% CI 2.4–54.6, *p* = 0.002; category 4: OR 5.9, 95% CI 1.4–23.5, *p* = 0.012). **Conclusions:** Patients on anticoagulation and those undergoing complex ERCP procedures (Schutz 3 or 4) are at particular risk of severe procedure-related complications.

## 1. Introduction

Since its introduction in 1968, Endoscopic Retrograde Cholangiopancreatography (ERCP) has become a cornerstone in diagnosing and treating pancreaticobiliary diseases [1]. Despite its minimally invasive nature, ERCP is associated with various complications, including pancreatitis, infections (such as cholangitis and cholecystitis), bleeding, perforation, and anaesthesia-related issues [2,3,4,5,6,7,8,9,10]. While numerous studies over the past two decades have identified patient- and procedure-related risk factors for post-ERCP complications [11,12,13,14,15,16,17,18,19], most of these complications are mild and self-limiting [2,5].

Severe post-ERCP complications, however, pose a significant challenge due to their association with increased morbidity and mortality. The current literature offers limited insight into the specific risk factors contributing to these severe outcomes. This study aims to fill this gap by identifying the key factors associated with severe post-ERCP complications. In this way, we hope to help endoscopists and referring physicians in their risk-benefit assessment with regard to ERCP for their patients.

## 2. Material and Methods

### 2.1. Patients

We conducted a retrospective cohort study using data from all ERCP procedures at Ghent University Hospital performed between January 2016 and December 2022, collected in a prospectively maintained ERCP register. The extent of prospectively collected data varied annually. The retrospective review of electronic patient files (EPD UZ Gent) and/or the prospective registry was used to gather patient characteristics, procedure details, complications, and severity. A Supplementary Table outlines variables collected prospectively or retrospectively (see Supplementary Table S1). Procedures deviating from classic ERCP, except for EUS-guided rendezvous, were excluded. When multiple complications occurred, only the most severe was analysed. For patients with multiple ERCPs, only the first procedure with a complication was included. The study was approved by the ethical committee (6 June 2023).

### 2.2. ERCP Procedure

Before the procedure, all patients provided written informed consent. ERCPs were performed by six endoscopists (1–30 years of experience) and three trainees (less than 1 year) supervised by the senior endoscopists. Patients received specific antithrombotic therapy instructions based on consensus guidelines [20]. New oral anticoagulants (NOACs) were stopped 48 h prior, Vitamin K antagonists (VKAs) 5 days prior (bridging with LMWH 3 days prior for high thromboembolism risk), Low-Molecular-Weight Heparins (LMWHs) 24 h prior, and P2Y12 inhibitors 5 days prior. Acetylsalicylic acid was not interrupted. Post-procedure, anticoagulants were resumed as per the guidelines, with exceptions in cases of complications like bleeding. All patients received prophylactic antibiotics and rectal NSAIDs to prevent post-ERCP infections and pancreatitis unless contraindicated.

All procedures were performed under general anaesthesia with intravenous propofol. Most cases used ‘monitored anaesthesia care’ (IV propofol without intubation). Hyoscine-n-butyl (Buscopan) was used, if needed, for muscle relaxation. Oxygen saturation, heart rate, and blood pressure were monitored automatically.

Standard care in the department involves first attempting a guidewire-assisted cannulation of the common bile duct (CBD) or pancreatic duct (PD). In case of unintentional PD cannulation, the guidewire was left in place and a second wire was used to cannulate the CBD (double-wire method). In case of failure, a precut technique was attempted. In case of failure despite precut with an urgent ERCP indication, an EUS-guided rendezvous procedure was performed during the same procedure. In less urgent cases, a reattempt was performed after an interval of at least 2 days, with an EUS-guided rendezvous procedure in case of a second failure.

The procedural report documented the indication, cannulation difficulty, findings, materials, interventions, technical success, and immediate complications, including their management.

The vast majority of patients were hospitalised for minimum one night. The morning after the procedure, each patient underwent a standard laboratory check, which included complete blood count, renal function, liver set, bilirubin, lipases, and CRP.

### 2.3. Definitions

We followed the 2020 European Society of Gastrointestinal Endoscopy (ESGE) guidelines on ERCP-related adverse events for complication definitions [6]. Sepsis was defined as life-threatening organ dysfunction due to a dysregulated host response to infection [21]. Procedure difficulty was classified using the modified Schutz criteria [22]. EUS-guided rendezvous procedures, performed routinely for only about 10 years, were not included in the Schutz classification (updated in 2011). In our study, these were classified as Grade 4 due to their technical complexity and because they are almost exclusively performed in tertiary centres by experienced ERCP endoscopists.

We graded the severity of ERCP-related complications using the Adverse Events in Gastrointestinal Endoscopy (AGREE) classification [23]. Complications with AGREE ≤ 2 were classified as “non-severe”, and those with AGREE > 2 as “severe” (see Supplementary Table S2).

### 2.4. Follow-Up

If no complications occurred, patients were discharged with the endoscopist’s approval. The hospitalisation report included the ERCP procedural details and post-procedural course. Upon discharge, patients were advised to seek medical help if new symptoms (e.g., abdominal pain or fever) arose. There was no standardised follow-up, but most patients had consultations that were scheduled based on individual needs and procedure success.

### 2.5. Study Objective

The primary study aim was to identify factors, both patient- and procedure-related, associated with the severity of the complications experienced by patients post-ERCP.

### 2.6. Selection of Variables

To assess potential factors linked to the severity of post-ERCP complications, we used risk factors from the recent ESGE guidelines [6] based on multiple studies. We also included factors for ‘severe’ post-ERCP complications from three prior studies, two retrospective (Cotten et al. [17], Kwak et al. [24]) and one prospective (Glomsaker et al. [25]) (see Supplementary Table S3). These factors covered general health (ASA 1–5 [26]) and procedure complexity (modified Schutz criteria 1–4 [22]).

Additionally, we analysed less-studied factors like time to cannulation, immunosuppressive therapy, ERCP endoscopist’s experience, and type of intervention. In total, 30 potential risk factors (17 patient-related, 13 procedure-related) were examined (see Supplementary Table S1).

### 2.7. Statistical Analysis

Categorical variables were analysed using Chi-squared and Fisher’s exact tests, and continuous variables with the Mann–Whitney U test (medians with IQR). Variables with a *p*-value < 0.20 in univariate analysis, plus risk factors from previous studies [17,24,25], were included in the multivariate logistic regression to identify independent risk factors for severe post-ERCP complications.

Goodness-of-fit was assessed using the Hosmer–Lemeshow test. The risk for severe vs. non-severe complications was expressed as an odds ratio (OR) with a 95% confidence interval (CI). Significance was set at *p*-value < 0.05. The Statistical Package for Social Sciences (SPSS, version 29.0, Chicago, IL, USA) was used for the statistical analysis.

## 3. Results

### 3.1. Patient and Procedural Characteristics

**Figure 1:**
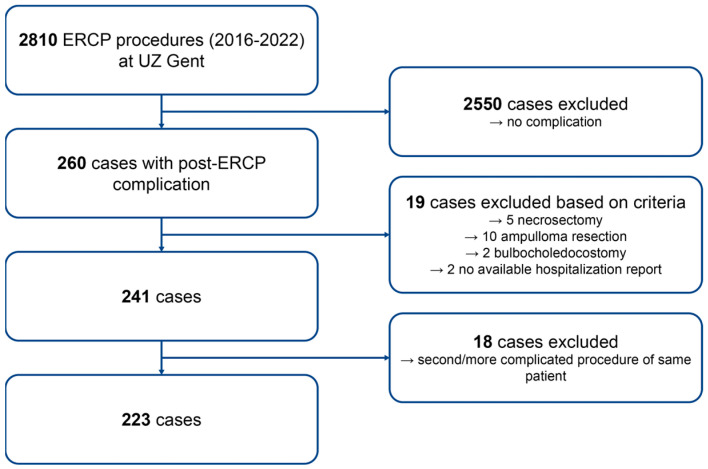
Flowchart of patient inclusion and exclusion.

Between January 2016 and December 2022, 2810 ERCP procedures were performed at UZ Ghent, with 241 (8.6%) resulting in post-ERCP complications after applying exclusion criteria. To avoid selection bias, only the first complication-related procedure per patient was included, leaving 223 complications (7.9%) for analysis (see Figure 1).

A total of 136 patients (60.9%) had major comorbidities, and 97 procedures (43.4%) were classified as complex (Schutz 3 or 4). The procedures were performed by nine endoscopists, including three trainees, with 91.5% (204 procedures) carried out by the three most experienced endoscopists. Patient and procedural characteristics are shown in Table 1.

### 3.2. Indications and Interventions Performed

Table 2 summarises the indications for ERCP; biliary obstruction was the most frequent indication (71.8%).

### 3.3. Complications

Table 3 outlines post-ERCP complications and their severity. Pancreatitis was the most common complication, followed by haemorrhage, infections, perforation, and cardiopulmonary failure. About 1 in 5 complications were classified as severe (49/241, 20.3%). The most common severe complication was haemorrhage (22/49 severe cases, 44.9%). Additional details about severe bleeding cases in our cohort, including endoscopic interventions and outcomes, are available in Supplementary Table S4. Although less common, perforation had the highest likelihood of resulting in a severe complication, with 66.7% (10/15) classified as severe. Six patients (6/2810, 0.21%) died within 30 days following the ERCP procedure. Five of them probably died from ERCP-related complications: two from cholangiosepsis with shock, two from bleeding, and one from perforation. Additionally, one patient experienced cardiopulmonary arrest during the procedure (see Supplementary Table S5).

### 3.4. Predictors of Severe Post-ERCP Complications

#### 3.4.1. Univariate Analysis

Univariate analysis identified four risk factors that increase the likelihood of experiencing a severe post-ERCP complication. Two of these risk factors are patient-related, including anticoagulative therapy (*p* = 0.055) and an age range of 65–79 years (*p* = 0.045). The other two risk factors are related to the procedure itself, including degree of difficulty (*p* = 0.002) and incomplete biliary drainage (*p* = 0.041). We refer to Table 4a,b and Table 5 for detailed results of the univariate analysis.

#### 3.4.2. Multivariate Analysis

Variables with a *p*-value < 0.20 in the univariate analysis and risk factors of “severe” post-ERCP complication, as identified in three previous studies [17,24,25], were included in a stepwise multivariate logistical regression analysis. This multivariate analysis identified two variables as independent risk factors of a severe post-ERCP complication as follows: one patient-related risk factor, i.e., anticoagulative therapy (OR 6.3, 95% CI 1.4–28.3, *p* = 0.016), and one procedure-related risk factor, specifically a high degree of difficulty according to Shutz’s categories 3 (OR 11.5, 95% CI 2.4–54.6, *p* = 0.002) and 4 (OR 5.9, 95% CI 1.4–23.5, *p* = 0.012). This is further detailed in Table 6.

## 4. Discussion

In this single-centre cohort study, we identified anticoagulative therapy and a high degree of procedural difficulty as independent risk factors associated with severe ERCP-related complications.

Our study reported a total complication rate of 7.9%, consistent with the 6.8% cited in a systematic review by Andriulli et al. [5]. Notably, 20.3% of these complications were classified as severe, aligning with the 24% reported by Andriulli et al., though the incidence of severe post-ERCP pancreatitis differed (5% in our study vs. 11% in theirs). This discrepancy is probably due to the fact that we did not classify prolonged hospitalisation as a severe adverse event, whereas Cotten et al. criteria [17] included prolonged stay exceeding 10 days as ‘severe’ in post-ERCP pancreatitis. Many patients in our cohort required extended hospital stays for reasons unrelated to the severity of pancreatitis, such as age and comorbidities.

The incidence of ERCP-related mortality was low at 0.21% (6 out of 2180), aligning with the expected outcomes [5]. Among the deceased, half had malignancies affecting the pancreatic or biliary tract, consistent with Glomsaker et al. [25].

Despite extensive research identifying the risk factors for post-ERCP complications [11,12,13,14,15,16,17,18,19], information to predict severe ERCP-related adverse events remains limited. Only three previous studies have examined these risk factors ([17,24,25]; see Supplementary Table S3), with poor general medical condition (ASA classification 3–5) identified as the sole common risk factor. Although a higher percentage of severe complications was observed in patients with an ASA classification of 3 or higher, this did not reach statistical significance in our analyses. Our study found that procedures rated as technically difficult (Shutz 3 + 4) significantly increased the risk of severe complications (OR 11.5 and OR 5.9, respectively), corroborating findings from Cotton et al. (OR 2.86) [17]. This underscores the necessity for careful evaluation before undertaking complex procedures and the importance of informing patients about their increased risk.

Our analysis also identified anticoagulant therapy (NOAC/VKA/LMWH) as a second independent predictor of severe complications, with 7 out of 12 severe cases in patients on anticoagulant therapy being haemorrhagic. Despite advancements in endoscopic techniques, including the use of fully covered metal stents for haemostasis, severe bleeding can still occur, leading to rapid haemorrhagic shock and subsequent ICU admission. In our cohort, two patients succumbed to haemorrhage, and three required ICU admission prior to any endoscopic intervention. This highlights the critical importance of recognising anticoagulation as a significant risk factor for severe post-ERCP bleeding, requiring prompt identification for earlier intervention, vigilant monitoring, and potentially more aggressive management strategies for these high-risk patients. Interestingly, antiplatelet therapy did not emerge as a significant risk factor, aligning with findings from the 2018 study by So Nakaji et al. [27].

Incomplete biliary drainage has been associated with a heightened risk of infectious complications [28,29]. Our study found that patients with incomplete drainage were more likely to experience severe complications, but this relationship did not hold up in the multivariate analysis.

The experience of endoscopists is expected to influence complication rates due to the procedure’s steep learning curve. While some studies confirm this correlation [30,31,32], others do not [11]. Our analysis did not find a significant link between endoscopist experience and severe complications, consistent with Cotton et al. [17]. Notably, we observed a low incidence of severe complications (5/39, 12.8%) among endoscopists with less than one year of experience, though this was not statistically different from more experienced colleagues. This may be attributed to the fact that less experienced endoscopists typically handle less complex cases, and in our centre, an experienced supervisor intervenes if cannulation is unsuccessful within 7 min.

Our study’s strength lies in its focus on the risk factors for severe post-ERCP complications in a large patient cohort. It provides a better insight into the risk/benefit ratio of ERCP based on procedural and patient-specific characteristics.

Our study has several limitations. We used a prospective registry of ERCP data, but it has to be taken in account that the consistency of prospectively gathered information varied annually, necessitating the retrospective analysis of patient records. Consequently, certain variables may be missing, compromising the robustness of our analysis.

Additionally, as a tertiary referral centre, our cohort may be biased towards more challenging and high-risk cases; for instance, 60.9% of our patients had significant comorbidities (ASA 3 or higher), double the prevalence found in comparable European studies [14,25,33].

Another limitation of our study is the variation in sample sizes across different patient categories in our univariate analysis (Table 4a,b). In some categories, the small number of cases limits the reliability of statistical estimates and requires cautious interpretation. Despite this limitation, our logistic regression model demonstrated satisfactory predictive accuracy, underscoring the relevance of examining a broad set of patient characteristics to capture potential associations with severe complications.

Moreover, while we identified 49 severe complications based on the AGREE classification, the clinical impact of these complications varies. Long-term morbidity associated with AGREE 3 post-ERCP pancreatitis can be more significant than the morbidity associated with AGREE 3 post-ERCP haemorrhage. Pancreatitis often entails prolonged hospitalisation, substantial pain, and complications related to third-spacing, which profoundly affect patient recovery and quality of life. In contrast, most AGREE 3 haemorrhage cases in our cohort required only short-term hospitalisation, with minimal pain and interventions, leading to quicker recoveries. This distinction emphasises the need for a nuanced understanding of severity within the AGREE framework.

Finally, our analysis did not account for any unreported or untreated complications occurring outside our institution, as we did not perform systematic follow-ups 30 days post-ERCP. This may have resulted in an underestimation of total complications and an overestimation of the proportion of severe cases.

In conclusion, our study indicates that the overall likelihood of severe complications after ERCP is relatively low, even in a tertiary centre handling complex cases in patients with significant comorbidities. Procedural difficulty and anticoagulative therapy were identified as independent risk factors for severe post-ERCP complications. Our findings warrant validation in larger prospective registries encompassing both academic and non-academic centres.

## Figures and Tables

**Table 1:** Patient and procedural characteristics.

**Patient Demographics and Characteristics**		**N**	**%**
Sex	Male Female	114 109	51.1 48.9
Age	<35 y 35–64 y 65–79 y >80 y	16 83 82 42	7.1 37.2 36.7 18.8
ASA	1 2 3 4	11 76 122 14	49.3 34.0 54.7 6.2
Comorbidities	Chronic pancreatitis End Stage Kidney Disease (ESKD) Thrombocytopenia Cirrhosis Primary Sclerosing Cholangitis (PSC)	24 2 13 6 6	10.8 0.9 5.8 2.7 2.7
Immunosuppressive therapy	Yes	40	17.9
Antithrombotic therapy	None Anticoagulant Antiplatelet	138 35 50	61.8 15.7 22.4
Previous ERCP	Yes	96	43.0
Previous post-ERCP pancreatitis (PEP)	Yes	10	4.5
**Procedural Characteristics**		**N**	**%**
Difficult cannulation	Yes	104	46.1
Time to cannulation (min)	<5 min 5–10 min >10 min	88 34 69	46.1 17.8 36.1
Precut sphincterotomy	CBD PD	32 7	14.3 3.1
Incomplete biliary drainage	Yes	31	15.9
Incomplete stone extraction	Yes	12	12.7
Bilirubin < 1 mg/dL	Yes	97	44.3
Non-dilated CBD	Yes	62	29.9
Degree of difficulty (modified Shutz criteria)	1 2 3 4	5 121 55 42	2.2 54.2 24.7 18.8
Endoscopist’s experience	<1 y 1–5 y >5 y	39 60 124	17.4 26.9 55.6

**Table 2:** Indications and type of interventions performed.

**Indication**	**n/N**	**%**
Bile duct stones	84/223	37.7%
(History of) pancreatitis	24/223	10.8%
Biliary cancer	25/223	11.2%
Bile duct leak	3/223	1.3%
Benign biliary stricture	51/223	22.9%
Pancreatic cancer	17/223	7.6%
Sphincter of Oddi dysfunction	1/223	0.4%
Ampullary stenosis	8/223	3.6%
Other	10/223	4.5%
**Type of Intervention**	**n/N**	**%**
Biliary stone extraction	70/223	31.4%
Biliary stricture treatment	101/223	45.3%
Pancreatic stricture treatment	15/223	6.7%
Pancreatic stone treatment	1/223	0.4%
EUS-assisted biliary drainage	25/223	11.2%
EUS-assisted pancreatic drainage	4/223	1.8%
Biliary leak	3/223	1.3%
Pancreatic leak	1/223	0.4%
Other	3/223	1.3%
Total (N)		100.0%

**Table 3:** Incidence of all post-ERCP complications and ‘severe’ post-ERCP complications per type of complication.

Complication Type	N ^ 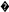 ^/223 (%)	Severe Complications (n ^‡^/N, %)			
		A3 ^§^	A4	A5	Total
Pancreatitis	82 (36.8%)	3 (3.7%)	1 (1.2%)	0 (0%)	4 (4.9%)
Infection (cholecystitis/ cholangitis/sepsis)	70 (31.4%)	5 (7.1%)	5 (7.1%)	2 (2.9%)	12 (17.1%)
Haemorrhage	55 (24.7%)	17 (30.9%)	3 (5.4%)	2 (3.6%)	22 (40.0%)
Perforation	15 (6.7%)	6 (40.0%)	3 (20.0%)	1 (6.7%)	10 (66.7%)
Cardiopulmonary failure	1 (0.4%)	0 (0%)	0 (0%)	1 (100.0%)	1 (100.0%)

^
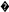
^ N = number of cases per type of complication. ^‡^ n = number of severe cases per type of complication. ^§^ A = AGREE classification.

**Table 4:** (**a**) Univariate analysis of patient-related risk factors for severe complications. Categorical variables. (**b**) Univariate analysis of procedure-related risk factors for severe complications. Categorical variables.

**(a)**				
**Variable**	**Category**	**n ^‡^/N ^ 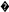 ^**	**%**	***p* Value ^§^**
Sex				0.507
	Male	23/114	20.2%	
	Female	26/109	23.9%	
Age				0.113
	<35 y	5/16	31.3%	0.354
	35–64 y	13/83	15.7%	0.080
	65–79 y	24/82	29.3%	0.045
	>80 y	7/42	16.7%	0.357
ASA classification				0.497
	1	1/11	9.1%	0.463
	2	14/76	18.4%	0.357
	3	30/122	24.6%	0.300
	4/5	4/14	28.6%	0.514
Previous pancreatitis				0.136
	Yes	6/44	13.6%	
	No	43/179	24.0%	
Previous post-ERCP pancreatitis (PEP)				0.695
	Yes	1/10	10.0%	
	No	48/213	22.5%	
No chronic pancreatitis				0.886
	Yes	5/24	20.8%	
	No	44/199	22.1%	
Previous ERCP				0.721
	Yes	20/96	20.8%	
	No	29/127	22.8%	
ESKD				0.000
	Yes	0/2 ^¶^	0.0%	
	No	49/221	22.2%	
Thrombocytopenia <50,000/mm^3^				1.000
	Yes	3/13	23.1%	
	No	46/210	21.9%	
Liver cirrhosis				0.343
	Yes	0/6	0.0%	
	No	49/217	22.6%	
PSC				0.122
	Yes	3/6	50.0%	
	No	46/217	21.2%	
Immunosuppressive therapy				0.176
	Yes	12/40	30.0%	
	No	37/183	20.2%	
Suspected SOD				1.000
	Yes	0/2 ^¶^	0.0%	
	No	49/221	22.2%	
Normal bilirubin < 1 mg/dL				0.227
	Yes	18/97	18.6%	
	No	31/122	25.4%	
Non-dilated CBD				0.105
	Yes	9/62	14.5%	
	No	40/152	26.3%	
Hilar obstruction				0.136
	Yes	10/31	32.3%	
	No	39/192	20.3%	
Antithrombotic therapy				0.159
	No	27/138	19.6%	0.269
	Anticoagulation	12/35	34.3%	0.055
	Antiplatelet	10/50	20.0%	0.703
**(b** **)**				
**Variable**	**Category**	**n ^‡^ /N ^ 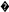 ^**	**%**	***p* Value ^§^**
Degree of difficulty (modified Shutz criteria)	Category			0.002
	1	0/5	0.0%	0.589
	2	16/121	13.2%	<0.001
	3	18/55	32.7%	0.026
	4	15/42	35.7%	0.017
Difficult cannulation				0.669
	Yes	24/104	23.1%	
	No	24/116	20.7%	
Time to cannulation (in min)				0.909
	<5 min	18/88	20.5%	0.658
	5–10 min	7/34	20.6%	0.831
	>10 min	16/69	23.2%	0.769
Pancreatic injection				0.279
	Yes	9/54	16.7%	
	No	40/169	23.7%	
Biliary precut sphincterotomy				0.988
	Yes	7/32	21.9%	
	No	42/191	22.0%	
Pancreatic sphincterotomy				0.352
	Yes	0/7	0.0%	
	No	49/216	22.7%	
Incomplete stone extraction				0.131
	Yes	5/12	41.7%	
	No	16/82	19.5%	
Incomplete biliary drainage				0.041
	Yes	11/31	35.5%	
	No	31/163	19.0%	
Endoscopist’s experience (in years)				0.276
	<1 y	5/39	12.8%	0.129
	1–5 y	13/60	21.7%	0.947
	>5 y	31/124	25.0%	0.222
Cholangioscopy				0.142
	Yes	5/12	41.7%	
	No	44/211	20.9%	
Balloon sphincterotomy				1.000
	Yes	3/15	20.0%	
	No	46/208	22.1%	
Intervention type				0.261
	Biliary stone extraction	16/70	22.9%	0.829
	Biliary stricture treatment	22/101	21.8%	0.950
	Pancreatic stricture treatment	1/15	6.7%	0.200
	Pancreatic stone treatment	0/1 ^¶^	0.0%	1.000
	EUS assisted biliary drainage	9/25	36.0%	0.072
	EUS assisted pancreatic drainage	0/4 ^¶^	0.0%	0.578
	Biliary leak	0/3 ^¶^	0.0%	1.000
	Pancreatic leak	1/1 ^¶^	100.0%	0.220
	Other	0/3 ^¶^	0.0%	1.000
Rendezvous procedure (EUS-assisted procedure)				0.260
	Yes	9/29	31.0%	
	No	40/194	20.6%	

^
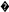
^ N = number of analysed procedures. ^‡^ n = number of ‘severe’ complications. ^§^ Statistical test used for categorical variables: Chi square test (or Fisher’s exact test where >20% of cells in the contingency table have expected frequencies < 5). ^¶^ Categories with small sample sizes (e.g., n < 5) may yield less reliable statistical estimates.

**Table 5:** Univariate analysis of risk factors for severe post-ERCP complication. Continuous variables.

Variable	Severe Complication	Non-Severe Complication	*p* Value ^‡^
	Median (IQR ^ 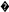 ^)	Median (IQR ^ 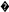 ^)	
Age	69.0 (56.0–76.5)	68.0 (54.0–76.3)	0.865
Time to cannulation in min ^§^	8.0 (2.0–18.5)	6.5 (3.0–13.0)	0.983

^
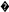
^ IQR = interquartile range. ^‡^ Statistical test used for continuous variables = Mann–Whitney U test. ^§^ Analysis based on 41/49 severe cases and 150/174 non-severe cases (due to missing data).

**Table 6:** Multivariate logistical regression analysis of patient- and procedure-related risk factors for severe complications.

Variable ^ 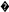 ^	Category	OR (95% Cl)	*p* Value
Antithrombotic therapy			0.051
	No	1.00 (Reference)	
	Anticoagulation	6.3 (1.4; 28.3)	0.016
	Antiplatelet		0.260
Age			0.995
	<35 y		0.916
	35–64 y		0.939
	65–79 y		0.833
	>80 y	1.00 (Reference)	
Degree of difficulty according to Shutz criteria			0.004
	1		
	2	1.00 (Reference)	
	3	11.5 (2.4; 54.6)	0.002
	4	5.9 (1.4; 23.5)	0.012
Incomplete biliary drainage	Yes/no		0.291

^
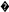
^ Selected variables for multivariate analysis = variables with a *p* value < 0.20 in the univariate analysis and identified risk factors related to severe complication from 3 previous studies [17,24,25].

## Data Availability

The data presented in this study are available on request from the corresponding author.

## Supplementary Materials

The following supporting information can be downloaded at: https://www.mdpi.com/article/10.3390/jcm13237481/s1, Table S1: Data collection methods by year: prospective and retrospective variables. Table S2: AGREE classification. Table S3: Studies on risk factors for ‘severe’ post-ERCP complications. Table S4: Severe haemorrhage cases: details. Table S5: Fatal cases: details.
