# Supplementary material for: Factors Associated with the Severity of ERCP-Related Complications: A Retrospective Single-Centre Study
Source: J Clin Med. 2024 Dec 9;13(23):7481. doi: 10.3390/jcm13237481 (PMC11642193; doi:10.3390/jcm13237481)
Supplement: Supplementary file 1 [file jcm-13-07481-s001.zip › jcm-3320319-supplementary.pdf]

SUPPLEMENTARY MATERIAL

Table S1. Data collection methods by year: prospective and retrospective variables.

**Variables**	**Years**			**Resource retrospective data**
	**2016-2018**	**2019-2020**	**2021-2022**	1. EPF/hospitalisation report<br>2. Check exact numbers in patients lab at time of ERCP<br>3. Procedural report<br>4.When not explicitly mentioned in the procedure report, the recorded images of the procedure were reviewed to determine and calculate the timing of cannulation and the diameter of the CBD<br>5. Endoscopist experience was calculated based on the name of the endosopist mentioned in the report
Sex	prospective	prospective	prospective	N/A
Age	prospective	prospective	prospective	N/A
ASA classification	retrospective	retrospective	retrospective	1
Previous pancreatitis	retrospective	retrospective	retrospective	1
Pevious post-ERCP pancreatitis (PEP)	retrospective	retrospective	retrospective	1
No chronic pancreatitis	retrospective	retrospective	retrospective	1
Previous ERCP	retrospective	retrospective	retrospective	1
ESKD	retrospective	retrospective	retrospective	2
Thrombocytopenia	retrospective	retrospective	retrospective	2
Liver cirrhosis	retrospective	retrospective	retrospective	1
PSC	retrospective	retrospective	retrospective	1
Immunosuppressive therapy	retrospective	retrospective	retrospective	1
Suspected SOD	retrospective	retrospective	retrospective	1
Normal bilirubin <1 mg/dL	retrospective	retrospective	retrospective	2
Non-dilated CBD	retrospective	retrospective	prospective	1,4
Hilar obstruction	retrospective	retrospective	prospective	1
Antithrombotic therapy	retrospective	retrospective	retrospective	1
Degree of difficulty (Modified Schutz criteria)	retrospective	retrospective	prospective	3
Time to cannulation (min)	retrospective	retrospective	prospective	3,4
Difficult cannulation	retrospective	retrospective	prospective	3
Pancreatic injection	retrospective	retrospective	prospective	3
Biliary precut sphincterotomy	retrospective	retrospective	prospective	3

Pancreatic sphincterotomy	retrospective	retrospective	prospective	3
Incomplete stone extraction	retrospective	retrospective	prospective	3
Incomplete biliary drainage	retrospective	retrospective	prospective	3
Endoscopist experience	prospective	retrospective	prospective	3,5
Cholangioscopy	retrospective	retrospective	prospective	3
Balloon sphincterotomy	retrospective	retrospective	retrospective	3
Intervention type	prospective	retrospective	prospective	1,3
Rendezvous procedure (EUS-assisted procedure)	prospective	retrospective	prospective	3

Table S2. AGREE classification.

**Grading**	**Definition**
No adverse event �	- A telephone contact with the general practitioner, outpatient clinic, or endoscopy service without any intervention or<br>- Extended observation of the patient after the procedure, <3 hours, without any intervention
Grade I	Adverse events with any deviation of the standard postprocedural course, without the need for pharmacologic treatment or endoscopic, radiologic, or surgical interventions.<br>- Presentation at the emergency ward, without any intervention or<br>- Hospital admission (<24 hours), without any intervention or<br>- Allowed therapeutic regimens are drugs as antiemetics, antipyretics, analgesics, and electrolytes or<br>- Allowed diagnostic tests: radiology and laboratory tests
Grade II	- Adverse events requiring pharmacologic treatment with drugs other than those allowed for grade I adverse events (i.e., antibiotics, antithrombotics, etc) or<br>- Blood or blood product transfusions or<br>- Hospital admission for more than 24 hours
Grade III<br>Grade IIIa<br>Grade IIIb	Adverse events requiring endoscopic, radiologic, or surgical intervention<br>Endoscopic or radiologic intervention<br>Surgical intervention
Grade IV<br>Grade IVa<br>Grade IVb	Adverse events requiring intensive care unit/critical care unit admission<br>Single-organ dysfunction (including dialysis)<br>Multiorgan dysfunction
Grade V	Death of the patient

� *Definition of adverse event: all negative outcomes for a patient that prevent completion of the planned procedure or cause any deviation from the standard postprocedural course.*

Table S3. Studies on risk factors for 'severe' post-ERCP complications.

**Author**	**Year of publication**	**Country**	**Setting**	**Study design**	**Cohort**	**RF for severe complication in multivariate analysis**
Cotton PB et al.(17)	2009	USA	Tertiary centre	Retrospective Monocentric	11497	-Poor health status (ASA III OR 2.38; ASA IV/V OR 7.65)<br>-Obesity (OR 5.18)<br>-Suspected/known biliary-duct stones (OR 4.08)<br>-Pancreatic manometry (OR 3.57)<br>-Complex procedures (Shutz grade 3) (OR 2.86)
Kwak et al.(23)	2020	USA	Tertiary centres	Retrospective Multicentric	1079	-Poor health status (ASA IV/V OR 3.13)<br>-Afro-Caribbean ethnicity (OR 2.57)
Glomsaker T. et al.(24)	2013	Norway	Mixed secondary and tertiary centres	Prospective Multicentric	2808	-Poor health status (ASA III OR 3.26; ASA IV/V OR 25.27)<br>-High age >90 yo (OR 4.36)<br>-Precut EST (OR 3.01)<br>->150 ERCPs annually (OR 1.74)

Table S4. Severe haemorrhage cases: details.

AGREE	Comment
3	RBPA, Post-papillotomy bleeding, endoscopic re-intervention with adrenaline injection and bicap
	Melena, Post-papillotomy bleeding, endoscopic re-intervention, adherent clot, no treatment
	Post-procedural anemia, post-papillotomy bleeding, endoscopic re-intervention with bicap
	Melena, Post-papillotomy bleeding, endoscopic re-intervention with adrenaline injection en bicap
	Post-procedural anemia, post-papillotomy bleeding, endoscopic re-intervention with bicap
	Post-procedural anemia, post-papillotomy bleeding, endoscopic re-intervention with bicap
	Post-procedural anemia, post-papillotomy bleeding, endoscopic re-intervention with placing metallic covered stent
	Post-procedural anemia, post-papillotomy bleeding, endoscopic re-intervention with adrenaline injection
	RBPA, endoscopic re-intervention, no active bleeding visualised
	Melena, old blood in duodenum, strong suspicion of post-papillotomy bleeding, adrenaline injection papilla
	Post-procedural anemia, post-papillotomy bleeding, endoscopic re-intervention with adrenaline injection
	Stenting malign biliary obstruction, post-ERCP melena, endoscopic re-intervention, old blood, no active bleeding, possible bleeding of the tumor
	Melena, endoscopic re-intervention with visualisation clotting between 2 plastic biliary stent, removal stent with rebleeding, placing metallic stent with successful hemostasis
	Post-procedural anemia, visible vessel next to papilla, endoscopic re-intervention with clipping visible vessel
	Melena, Post-papillotomy bleeding, endoscopic re-intervention with adrenaline injection and clipping
	Melena, old blood in duodenum, strong suspicion of post-papillotomy bleeding, adrenaline injection papilla
	Melena/rbpa, post-papillotomy bleeding, radiological intervention with embolization
4	Melena, Post-papillotomy bleeding, haemorrhagic shock with transfer UCI. After stabilisation endoscopic reintervention with successful hemostasis.
	Hematemesis with haemorrhagic shock, requiring transfer to the intensive care unit. Bleeding due to a pseudo-aneurysm, necessitating radiological intervention with embolization.
	Post-papillotomy bleeding, haemorrhagic shock with transfer UCI. After stabilisation endoscopic reintervention with successful hemostasis.
5	Haemorrhagic shock, admission ICU, eventually death (see supplemental table 5 for further details)
	Haemorrhagic shock, admission ICU, eventually death (see supplemental table 5 for further details)

Table S5. Fatal cases: details.

Case	Relation ERCP with death	Post-ERCP complication	Age	Serious comorbidity	Details occurred complication	Comment/remark
1	Probable	Cholangiosepsis with shock	89	No	ERCP was performed due to suspected obstructive choledocholithiasis. The day following the ERCP, the patient developed septic shock, requiring vasopressor support. This progressed to multiple organ failure, and in consultation with the family, therapy was discontinued after one day of intensive care admission	A mildly elevated CRP was present prior to the ERCP, prompting the question of whether cholangitis had already been developing before the procedure. Despite this, blood cultures remained negative.
2	Possible	Cholangiosepsis with shock	87	Yes (CHF, DM)	ERCP was performed due to suspected obstructive choledocholithiasis. The day following the ERCP, the patient developed septic shock, requiring vasopressor support.Progressed to MOF with massive aspiration, leading to asystole. Resuscitation was stopped after 8 minutes	In the days prior to the ERCP, the patient had been treated for cholangitis with antibiotics, resulting in a successful reduction of inflammatory markers before the procedure.
3	Probable	Cardiopulmonary failure	78	Yes (Metastatic cholangiocarcinoma)	During the ERCP, prior to successful CBD cannulation and after one failed attempt, the patient developed ventricular fibrillation with circulatory arrest. Resuscitation efforts were discontinued after 20 minutes due to lack of success.	Ventricular fibrillation may have been triggered by anesthesia; however, anesthesia is essential for performing the procedure.
4	Probable	Bleeding	72	Yes (Kidney transplant)	The ERCP was performed for obstructive jaundice due to a newly diagnosed pancreatic head tumor. During ERCP after unsuccessful cannulation and a false passage, bleeding occurred but initially stopped spontaneously during the procedure. The patient then developed hemorrhagic shock, requiring unsuccessful embolization by interventional radiology. Surgical intervention via open laparotomy and an attempt at anticoagulation also failed, leading to deterioration of hemorrhagic shock and eventual death.	
5	Probable	Perforation	63	Yes (Metastatic pancreascarcinoma)	The ERCP was performed due to obstructive jaundice in a patient with pancreatic carcinoma. After unsuccessful CBD cannulation using both precut and double wire techniques, the procedure was	Had the patient been in better overall condition, a decision for surgical repair of the perforation

					converted to an EUS-guided rendezvous technique, which also failed. Following the procedure, the patient developed an acute abdomen with duodenal perforation. Although surgical intervention was indicated, a decision was made in consultation with surgery and the family to pursue a comfort care approach due to the poor prognosis and the patient's overall condition.	would likely have been made, which could have potentially saved the patient.
6	Probable	Bleeding	56	Yes (Metastatic HCC)	The ERCP was performed for obstructive jaundice due to newly diagnosed metastatic HCC at the liver hilum, involving papillotomy, balloon dilation, and placement of two biliary stents. During the procedure, bleeding occurred but was initially controlled. Post-procedure, the patient developed melena, leading to another endoscopy that revealed a clot at the papilla without active bleeding. Persistent melena progressed to hemorrhagic shock, requiring ICU transfer. Stabilization was difficult, and in consultation with the family, a decision was made to pursue comfort care due to the metastatic condition.	Had the patient had a better prognosis, a new intervention (endoscopic, radiological, or surgical) would likely have been considered to address the bleeding, which could have potentially saved the patient. However, it is uncertain whether stabilization could have been achieved first.
